# Ethical Use of Artificial Intelligence in Research Publication: A Scoping Review

**DOI:** 10.34172/ijhpm.9759

**Published:** 2026-08-26

**Authors:** Sara Dakhesh, Shahnaz Khademizadeh, Rasha Atlasi, Mojgan ZareiVenovel, Ali Ouchi

**Affiliations:** ^1^Department of Medical Library and Information Sciences, School of Allied Medical Sciences, Tehran University of Medical Sciences, Tehran, Iran.; ^2^Department of Knowledge and Information Science, Faculty of Educational Sciences and Psychology, Shahid Chamran University of Ahvaz, Ahvaz, Iran.; ^3^Endocrinology and Metabolism Research Center, Endocrinology and Metabolism Clinical Sciences Institute, Tehran University of Medical Sciences, Tehran, Iran.; ^4^Social Determinants of Health Research Center, Lorestan University of Medical Sciences, Khorramabad, Iran.; ^5^Department of Medical Library and Information Science, School of Management and Medical Informatics, Tabriz University of Medical Sciences, Tabriz, Iran.

**Keywords:** Generative Artificial Intelligence, Large Language Models, Scientific Integrity, Research Ethics, Scientific Misconduct

## Abstract

**Background:**

Artificial intelligence (AI) is increasingly used in research and scientific publishing, improving efficiency. However, it raises ethical concerns regarding scientific integrity, authorship, and originality. This study aimed to identify the main ethical challenges of AI use in research publishing and to explore factors influencing its responsible use.

**Methods:**

This scoping review followed Arksey and O’Malley’s framework and PRISMA-ScR (Preferred Reporting Items for Systematic reviews and Meta-Analyses extension for Scoping Reviews) reporting guidelines. The review addressed two questions on the ethical challenges associated with AI use in research publishing and the factors influencing its ethical application. A comprehensive search was performed in PubMed, Web of Science, Scopus, and Embase, supplemented by additional searches. Studies eligible for inclusion were original research articles published up to January 2025 with accessible full texts. Two reviewers independently screened the records and charted the data; disagreements were resolved through consultation with a third reviewer. Extracted data included study characteristics, aims, design, and key findings.

**Results:**

The search retrieved 20 952 records, of which 20 were included after screening. The analysis identified 10 key ethical challenges for the use of AI in research publishing, which broadly relate to three overarching concerns: threats to scientific integrity, challenges to transparency and accountability in AI‑assisted research processes, and governance gaps in policy and regulatory frameworks. Furthermore, factors influencing ethical AI use were grouped into 10 dimensions across individual, institutional, and structural levels that shape researchers’ behavior and publishing practices.

**Conclusion:**

The responsible use of AI in scientific publishing requires a shift from ad hoc approaches to an integrated ethical, technical, and legal governance system. Since health policies and clinical guidelines rely on published evidence, the ethical integration of AI in scientific publishing is essential to ensure the reliability and transparency of health research. This study highlights the necessity of developing policies, regulatory frameworks, and ethical standards in this area.

## Background

 In recent decades, rapid technological advances have transformed scientific research and knowledge production. A key motivator of this change is the growing use of artificial intelligence (AI), particularly large language models (LLMs) such as ChatGPT. These systems can generate and analyze natural language with a level of precision and coherence that was previously unattainable.^1-4^ ChatGPT reached millions of users within days and exceeded 100 million users within two months.^5^ These tools are now widely used in research, writing, data analysis, and scholarly assessment.^6,7^ Their rapid uptake is partly due to ChatGPT being the first LLM freely available to the public.^8^

 AI has increased scientific productivity but also raises questions about research ethics, integrity, and accountability.^1,2^ These tools use deep learning and natural language processing to help with idea generation, literature reviews, and manuscript drafting.^9-11^ While AI can reduce workloads, it blurs the line between human and machine-generated content. AI has challenged traditional concepts of authorship and originality.^12^ A major concern is whether AI-generated outputs can be considered ethically and scientifically valid knowledge.^13,14^

 In the AI era, ethical issues in scholarly publishing have received increasing attention.^15,16^ Honesty, transparency, and integrity remain core principles of research ethics. However, generative AI has created a need to reconsider these principles.^17,18^ AI-assisted writing raises several ethical and conceptual questions. These include issues related to authorship attribution and the appropriate level of disclosure regarding AI involvement. Another question concerns whether AI should be regarded merely as a tool supporting the writing process.^19^ AI can support different stages of research, from hypothesis generation to manuscript preparation.^20^ However, it still has problems with accurate referencing.^21^ The use of AI-generated content in academic work may threaten scholarly integrity and has been linked to increased student plagiarism.^22,23^ These issues show the need to re-examine traditional ethical norms in AI-assisted research.^24^

 Researchers increasingly use LLMs for writing, editing, abstract generation, and preliminary data analysis.^25,26^ These tools improve efficiency. However, they may produce hallucinations and unreliable content. This risk increases the need for human oversight.^27,28^ Their misuse may also result in fraudulent or duplicate publications. Such practices threaten the integrity of the scholarly evaluation system.^29-31^

 Beyond publishing, AI raises broader ethical and philosophical concerns.^32,33^ AI systems are becoming more autonomous and capable of learning. This development has led scholars to question whether AI could have moral agency. It has also prompted debate regarding the need for appropriate ethical and legal frameworks.^32,34-37^ These debates remain largely theoretical. Nevertheless, they indicate that AI is not only a research tool. It is also a subject with ethical and philosophical implications for scientific practice.^32^

 AI has complicated the notion of research integrity in scholarly publishing.^38,39^ Integrity now requires transparency about how AI tools are used. It goes beyond avoiding plagiarism or data fabrication. Many journals have issued guidelines on this issue.^40-43^ They emphasize disclosing AI use. They require humans to remain responsible for the content. They also state that AI systems should not be listed as authors. However, accurately determining the extent of AI contribution remains challenging in practice.

 Recent discussions highlight the need for clearer reporting of AI use. Transparency should go beyond simple binary disclosures. It should not be limited to stating whether AI was used. Current standards instead recommend structured reporting frameworks. These frameworks organize AI use around specific research tasks. One example is the Generative AI Delegation Taxonomy (GAIDeT). This framework classifies how research tasks can be delegated to AI across domains such as conceptualization, data management, and manuscript preparation. Such taxonomies require detailed reporting of AI involvement. Researchers must specify the task performed by AI, the extent of AI contribution, and the level of human oversight. This approach supports research integrity.^44^

 AI in scholarly publishing also has broader social implications. It can help democratize knowledge. For example, it may support researchers in developing countries or non-English speakers by improving their writing and visibility.^45^ However, excessive dependence on LLMs can potentially hinder creativity. Additionally, it may encourage similar patterns of thought and create new inequalities in access.^46^ This tension shows that the ethics of AI use in scholarly publishing must address more than individual researcher practices. It must also consider institutional and structural issues.^47^

 Several recent studies have examined the role of AI, especially LLMs such as ChatGPT, in scholarly publishing. Mijwil et al reported that ChatGPT challenges traditional ideas of human authorship and calls for reconsidering research ethics.^1^ Ugwu et al, in a rapid review, highlighted the need for clear guidelines to disclose AI use in writing.^28^ Bhargava et al also emphasized the importance of human accountability frameworks to protect scientific credibility.^19^ In contrast, Sharma noted that AI can help democratize knowledge production and support non-English-speaking researchers.^45^ Overall, these studies suggest that AI may improve writing and access, but it also raises new ethical challenges for research integrity and requires updated policies and guidelines.

 Despite growing interest in AI use in scholarly publishing, the evidence remains fragmented. No comprehensive review has mapped the ethical challenges and the factors that influence responsible use. These issues are particularly important in the health sciences. Published research in this field informs clinical guidelines and health policy decisions. Therefore, research must be credible and transparent. This is essential to maintain trust in health evidence and support evidence-based decision-making.

 Given this gap, a focused review is needed. This scoping review aims to map the existing literature by addressing two questions:

- **RQ1)** What ethical challenges are associated with the use of AI in publishing research outcomes?
- **RQ2)** What factors shape the responsible and ethical use of AI in scholarly publishing?

## Materials and Methods

 This scoping review followed the framework proposed by Arksey and O’Malley.^48^ Its reporting followed the PRISMA-ScR (Preferred Reporting Items for Systematic reviews and Meta-Analyses extension for Scoping Reviews) guideline.^49^ Scoping reviews are suitable for mapping knowledge across different types of studies.^50^ They can also include multiple types of evidence.^51^

###  Identifying the Research Question

 The present study aims to examine two main research questions: First, what ethical challenges are associated with the use of AI in publishing research outcomes? Second, what factors shape the responsible and ethical use of AI in scholarly publishing?

###  Identifying Relevant Studies

 The search strategy was developed on January 27, 2025. It was based on key concepts and Medical Subject Headings (MeSH). We consulted two subject-matter experts. Their expertise included research and publication ethics and the use of AI tools and LLMs. Based on their input, we selected the keywords and organized them into two groups.

 The first category covered concepts related to AI, chatbots, machine learning, and similar technologies. The second category addressed ethical concepts, including research integrity, misconduct, and key stakeholders in the publication process, such as researchers, students, authors, reviewers, editors, and policy-makers. Search syntaxes were then tailored for each database. These databases included PubMed, Web of Science, Scopus, and Embase. The detailed search strategies for all databases are presented in Supplementary file 1. The Web of Science search strategy is shown below, where “TS” refers to the topic field.

 T**S=(**(Intelligence AND Artificial) OR (Computer AND Reasoning) OR (Machine AND Intelligence) OR (Computational AND Intelligence) OR (Computer AND Vision AND System *) OR (“Chat Bot” OR “Chat Bots” OR ChatBot OR ChatBots OR “AI Chat” OR GPT OR “Generative Artificial Intelligence” OR “Chat GPT” OR ChatGPT OR ChatGPTs)** AND **((Fraud * AND Scientific) OR (Fraudulent AND Data) OR (Research AND Misconduct) OR (Scientific AND Dishonest *) OR (academic AND misconduct) OR (Academic AND Integrity) OR (Research AND Integrity) OR (Scientific AND integrity) OR (Academic AND honesty) **OR** ((Ethic * OR Moral OR misconduct OR Integrity) AND (Publish OR Publishing OR Publication * OR Writing * OR write OR draft * OR Composition OR author * OR Academic OR Student * OR Researcher * OR Professor * OR (Faculty AND member *) OR Publisher * OR Editor * OR Reviewer * OR Policymaker *))**))**

 Following the search, 20 952 records were identified. All records were exported to EndNote (version 20). We removed 7690 duplicate records and 360 retracted articles. This left 12 902 records for screening.

 During title and abstract screening, 410 potentially relevant articles were identified. 16 full texts were not available. The remaining 394 articles were assessed in full text. At this stage, 23 review articles and 45 items such as books, short reports, conference papers, editorials, letters to the editor, perspectives, news articles, and commentaries were excluded. In addition, 313 articles did not meet the study objectives. Finally, 13 original research articles were included as the final records.

 In parallel, we conducted supplementary searches. We manually screened the websites of key journals identified in the initial database search. We aimed to find additional relevant studies.

 We also searched Google Scholar using keywords related to AI, research ethics, and scientific publication. Retrieved records were screened by title and abstract based on the eligibility criteria. In addition, we reviewed the academic profiles of active researchers in this field to identify further publications.

 Two reviewers conducted the supplementary searches independently. A third reviewer evaluated the retrieved records and the selection decisions to ensure consistency with the study objectives. We also performed backward citation chaining by screening the reference lists of articles identified in the initial search.

 These supplementary searches identified 135 records. After title and abstract screening, 74 records were considered potentially relevant. The full texts of accessible and eligible articles were assessed. This process resulted in 35 records. We then excluded 5 duplicates, 15 review articles, and 8 editorials or letters. Finally, 7 original research articles were included from the supplementary searches. These 7 articles were combined with the 13 articles identified in the database search. In total, 20 records were included in the review (Figure).

**Figure:**
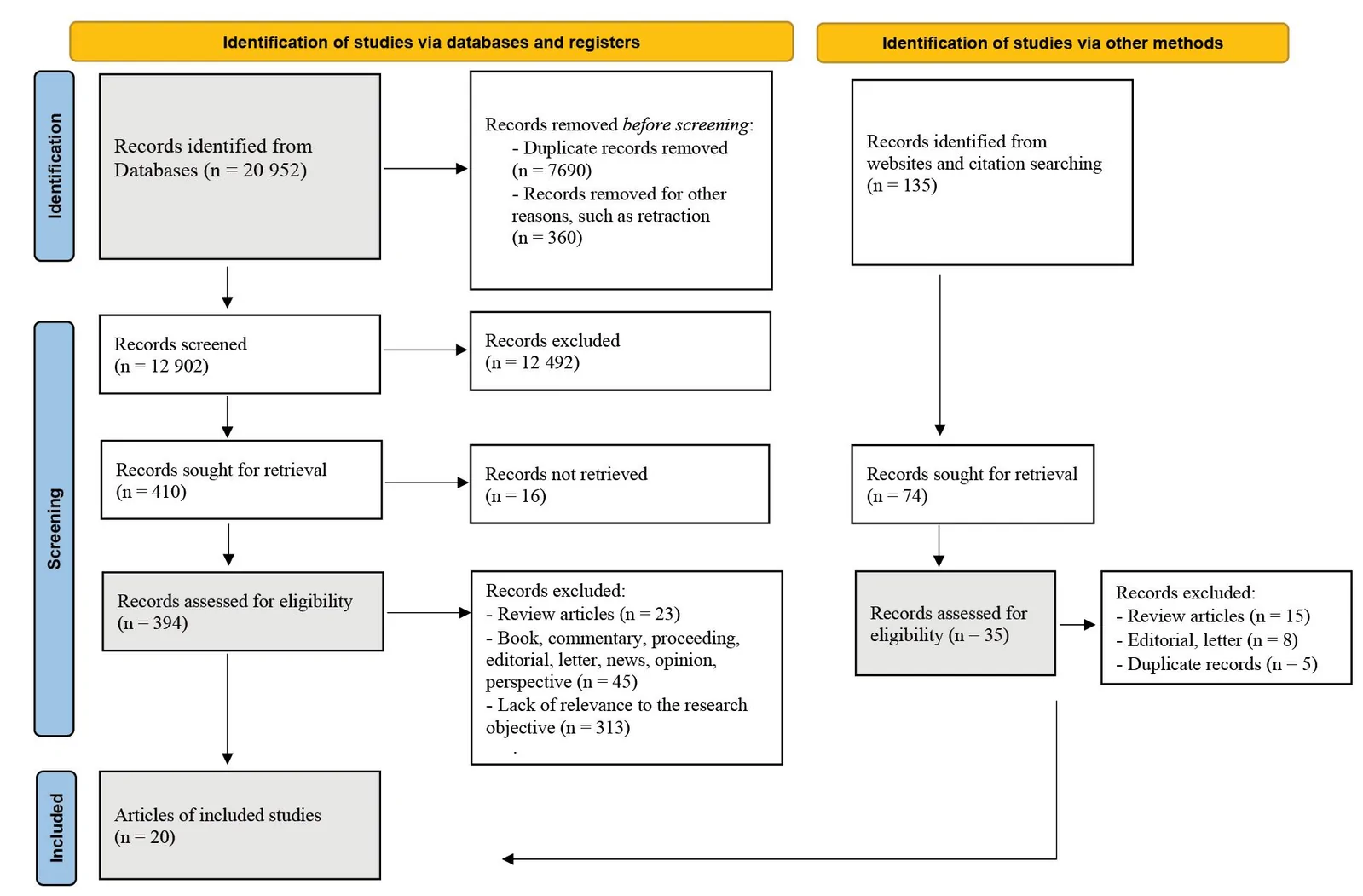
PRISMA Flow Diagram of the Literature Screening Process. Abbreviation: PRISMA, Preferred Reporting Items for Systematic reviews and Meta-Analyses.

###  Study Selection

 During the eligibility assessment, two researchers independently screened the titles and abstracts. They also reviewed the full texts of potentially relevant studies. Any disagreements were resolved by a third researcher.

 At the initial stage, a wide range of publication types was considered. These included policy statements, publisher guidelines, editorial policies, consensus statements, and reporting frameworks. However, after reviewing a sample of these sources, we found that the extracted themes largely overlapped with those from original studies. After consultation with experts in review methodology, we decided to restrict the review to original research articles. This decision was made to strengthen the credibility and consistency of the evidence base.

 The inclusion criteria were studies that examined publication ethics in AI-assisted research and were aligned with the objectives of this review. Eligible studies were published up to January 2025. Only records with accessible full texts (PDF) were included. Publications in all languages were considered.

 The exclusion criteria included studies that did not address the research questions or had no accessible full text. We also excluded review articles, books, short reports, conference papers, editorials, letters to the editor, perspectives or viewpoints, news items, and commentaries.

###  Charting the Data

 The next stage involved charting the data. Two reviewers independently extracted the data using a standardized data charting form. The extracted items included authors, year of publication, country, study aim, study design, and key findings.

 To enhance methodological rigor, the charting form was piloted on five randomly selected included studies. Then, minor revisions were made to refine the variables. The charting process was designed to align with the review questions and the predefined inclusion criteria.^52^

 Any disagreements were resolved through discussion and consensus. If needed, a third senior reviewer was consulted.

 Data charting followed a mixed coding approach. Deductive coding was applied based on the predefined research objectives. Inductive coding was used to identify emerging ethical themes. Codes were generated from concepts identified in the text. Related codes were then grouped according to similarities and differences. These groups were labeled to form descriptive themes, and broader themes were subsequently identified.

 Finally, the findings were synthesized into two main categories: ethical challenges and factors influencing the responsible use of AI tools in research.

###  Collating, Summarizing, and Reporting the Results 

 The quality of the included studies was assessed using the Mixed–Methods Appraisal Tool and the Joanna Briggs Institute checklist. Two reviewers independently conducted the assessment.

 The Mixed-Methods Appraisal Tool allows the quality appraisal of different study designs using a single tool. It includes criteria for quantitative, qualitative, and mixed-methods studies.^53^ Any disagreements between the reviewers were resolved through discussion. When consensus was not reached, a third reviewer made the final decision.

 The appraisal examined the clarity of the study objectives, the appropriateness of the methodology, the relevance of the findings, and the rigor of the conclusions. Additional details are presented in the Results section.

## Results

 A total of 20 952 records were initially identified through database searches. After removing duplicates and applying the inclusion and exclusion criteria, 20 articles were deemed eligible for the review. Most of these articles were published between 2023 and 2025. Researchers from the USA contributed the largest number of studies (n = 6; Table 1). To ensure conceptual clarity, this study distinguished between two concepts: “ethical challenges” and “influencing factors.” Ethical challenges refer to the risks, dilemmas, or ethical concerns arising from the use of AI in research publishing. Influencing factors are the systemic, organizational, or procedural conditions that can mitigate these challenges.

**Table 1:** Characteristics of the Included Studies

**Author(s) **	**Year** ^a^	**Country** ^b^	**Aim**	**Study Design**	**Key Findings**
Resnik and Hosseini^54^	2025	USA	Examine AI’s impact on the ethics of science. Develop new guidance for the appropriate use of AI. Focus on what is commonly referred to as narrow (or weak) AI.	Qualitative - Content analysis	While AI offers significant benefits for research, it also presents complex ethical challenges, highlighting the necessity for established principles governing its responsible use.
Da Veiga^55^	2025	South Africa	Proposed guidelines for AI research ethics in scientific publishing to inform coherent and consistent policies among publishers and academic institutions.	Qualitative - Content analysis	An examination of publishers’ policies reveals considerable heterogeneity in guidelines governing the use of AI in scholarly publishing. This study highlights the need to establish unified ethical standards and clarify existing policies.
Lei et al^56^	2025	China	Investigated the ethical implications of generative AI in academic publishing, with particular attention to user agreements.	Quantitative - Survey	User agreements associated with generative AI models are integral to the ethical landscape of scholarly publishing, as they often implicitly delineate limitations and responsibilities. Consequently, greater scrutiny of these agreements by regulators and researchers is essential to ensure the ethical application of AI.
Farangi et al^57^	2025	China	Researchers’ ethical concerns about the deployment of generative AI in research and their emotional responses.	Qualitative - Content analysis	Future research should place greater emphasis on ethical issues and emotional reactions to generative AI, as these factors play a pivotal role in guiding the responsible use of such technologies.
Agbor et al^58^	2024	Nigeria	Reflected on the implications of AI adoption in academic research, examining its potential benefits and threats.	Qualitative - Content analysis	Although AI offers promising benefits for academic work, excessive reliance on it may undermine scholarly skills and critical thinking, leading to academic indolence over time.
Chetwynd^59^	2024	USA	Use of AI in scientific writing.	Qualitative - Content analysis	Although AI contributes to greater efficiency and equity in scholarly publishing, ensuring the precision and up-to-date nature of generated content requires active engagement from researchers.
Giray et al^60^	2024	Philippines	Examined the transformative impact of ChatGPT integration in scientific research.	Qualitative - Content analysis	ChatGPT has the potential to fundamentally transform the way knowledge is structured, retrieved, analyzed, and shared in scientific research. Nevertheless, its capacity to truly impact scientific inquiry depends on deliberate usage and ongoing supervision.
Akila and Mohanbabu^61^	2024	India	Suggested strategies to support transparency, accountability, and responsible research practices.	Qualitative - Content analysis	The responsible development of AI depends on collaboration among researchers, ethicists, policy-makers, and society as a whole. This process necessitates a shared dedication to ethical standards, with ethical research and publication serving as a cornerstone for AI progress.
Lund and Naheem^62^	2024	USA	Examining the AI authorship policies of 300 top academic journals.	Qualitative - Content analysis	Most journals do not permit AI authorship but allow its use as a cited writing tool. These evolving policies are vital for maintaining trust in scientific and scholarly publishing.
Lehr et al^63^	2024	USA	Probed the capabilities of GPT-3.5 and GPT-4 across four central components of the scientific process: as a research librarian, research ethicist, data generator, and novel data predictor, using psychological science as a testing field.	Quantitative - Empirical	Despite its strengths in assisting with research tasks, ChatGPT (GPT-4) has limitations in critical evaluation and citation accuracy, underscoring the need for human oversight and adherence to ethical frameworks to maintain research integrity.
Sobaih^64^	2024	Egypt	Perceptions of academics and their leaders regarding the use of AI chatbots in research and publication.	Qualitative - Phenomenological	The extensive use of AI chatbots in research raises ethical concerns that may contribute to the proliferation of pseudoscience in developing countries.
Sysoyev^23^	2024	Russia	Examined students’ understanding of authorship ethics and AI-related plagiarism through questionnaire development and an online survey.	Quantitative - Survey	Teaching students about authorship ethics through faculty guidance, alongside clear institutional frameworks for the use of AI in academic and research tasks, plays a crucial role in mitigating AI-related academic misconduct.
Akpur^65^	2024	Turkey	Investigated the potential of ChatGPT to assist with academic writing tasks.	Quantitative - Empirical	ChatGPT is not a substitute for human creativity and intellect in academic work; nevertheless, it can function as a valuable aid in idea generation and data identification.
Pinchuk and Malytska^66^	2024	Ukraine	Analyzed the literature on plagiarism prevention, AI in research culture, academic integrity in universities, and changes in the assessment of student achievement.	Qualitative - Content analysis	AI may support research and publication, but without proper oversight and ethical frameworks, it can contribute to plagiarism, data distortion, and threats to scientific integrity.
Moffatt and Hall^67^	2024	USA	Analysis of ethical issues related to the use of new AI tools in manuscript writing.	Qualitative - Content analysis	Rather than prioritizing efficiency and rapid output alone, the future of scientific inquiry must be anchored in principles of quality, fairness, responsibility, and human integrity. This highlights the need for well-defined policies, transparency, researcher education, and collective commitment to the ethical use of AI.
Hosseini et al^68^	2023	USA	Discuss ethical issues related to using and disclosing AI tools, such as ChatGPT and other systems based on LLMs, to write or edit scholarly manuscripts.	Qualitative - Content analysis	The application of LLMs, including ChatGPT, in writing, reviewing, and editing scientific manuscripts introduces significant ethical dilemmas for scholars and journals. We argue that banning LLMs is counterproductive, as it is unlikely to be enforceable and may encourage the use of opaque or unreported methods.
Baloğlu and Çakalı^69^	2023	Turkey	Examines the effects of using artificial intelligence tools in academic research on academic ethics.	Qualitative - Content analysis	The deployment of AI tools in academic research carries the risk of ethical breaches, and the scarcity of tools capable of identifying such misconduct poses a major challenge.
Bahammamet al^70^	2023	Saudi Arabia	Examines the benefits and risks of LLMs and AI and the need for guidance on their responsible use.	Qualitative - Content analysis	Through expanded discussion and the creation of well-defined frameworks, it is possible to support responsible AI and LLM use while optimizing advantages and mitigating potential harms.
Ryan and Stahl^71^	2021	Sweden	Clarifies ethical principles guiding the development and use of AI systems.	Qualitative - Content analysis	While ethical principles are largely consistent across guidelines, putting them into practice is challenging. This research identifies key ethical frameworks to support developers and users in applying AI ethics in a meaningful and enforceable manner.
Hagendorff^72^	2020	Germany	Examines the implementation of ethical principles in the research, development, and application of AI systems, and ways to improve the effectiveness of AI ethics.	Qualitative - Content analysis	AI ethical guidelines are often symbolic and weakly enforced, highlighting the need for virtue ethics approaches and institutional reforms to achieve real ethical impact.

Abbreviations: LLM, large language model; AI, artificial intelligence. ^a^ The studies were sorted according to year of publication. ^b^ The country of each study was determined according to the corresponding author’s affiliation.

###  Ethical Challenges of AI Use in Research Publishing

 This review identified ethical challenges associated with AI in research publishing, categorizing them into 10 main dimensions with 43 specific items (Table 2). These dimensions cover issues such as scientific integrity, knowledge production quality, accountability, intellectual property, transparency, algorithmic bias, data security, and research policy.

**Table 2:** Ethical Challenges of Using Artificial Intelligence Technology in Research Publication

**No.**	**Dimensions**	**Challenges**
1.	Promoting pseudoscience and distorted knowledge	- Generation of fabricated data, evidence, and hallucinations without support from reliable sources^54,55,58,60,62,63,65,69,70^
		- Creation of fake references and citations^60,62,64,65,69^
		- Fabrication or distortion of quotations^64,70^
		- Generation of coherent but false and misleading scientific-style narratives^60,64^
		- Misinterpretation of previous studies due to a lack of precision in evaluating research evidence^62,65^
		- Incorrect paraphrasing of scientific concepts and texts due to concept drift or gradual changes in knowledge representation by language models^60,63^
2.	Violation of research originality	- Risk of machine-mediated plagiarism, commonly referred to as “AIgiarism^a^”^23,58,59,63,65,66,69^
		- Increase in self-plagiarism and covert plagiarism through non-transparent paraphrasing of content^66,69^
		- Blurring of boundaries between human-generated and AI-generated content due to the limited effectiveness of current detection tools^54,69^
		- Potential rise in scientific misconduct among researchers as AI tools continue to advance^58,66^
3.	Undermining creative and critical thinking	- Reduction in research innovation and creativity due to repetitive research suggestions^58,65,67,70^
		- Diminished motivation for independent thinking and idea generation^57,58,60,67^
		- Cognitive dependency arising from excessive reliance on AI tools^58,59^
		- Decline in language proficiency and research skills^57^
		- Replacement of in-depth analysis by surface-level interpretation in research^60,64^
4.	Ambiguity in intellectual property	- Uncertainty regarding ownership of AI-generated scholarly content^23,58,60,62,64,65,67^
		- Ineligibility of AI tools for authorship due to their inability to assume scholarly responsibility^62,63,67,68^
		- Blurring of boundaries between researchers’ roles and AI functions in the production of scholarly texts^63,68^
		- Lack of accountability for scientific and ethical errors^56,60,67,70,72^
		- Threats to publishers’ and original authors’ rights resulting from unauthorized reproduction and dissemination of texts^23,65^
5.	Threat to scientific integrity	- Lack of transparency in the application of AI tools during the research process^55,59,62,66,70^
		- Discrepancies in disclosure policies among journal publishers^54,55,62,71^
		- Undermining research integrity due to disregard for ethical codes^23,66,67^
		- Ambiguity in AI tool user agreements leading to user confusion^56^
		- Challenges in accurately tracking AI contributions in the research process^55,66^
6.	Bias and discrimination in text generation	- Generation of biased outputs in text rewriting and publication^55,60,61,63,65,70,72^
		- Production of texts reflecting cultural and gender stereotypes due to latent biases^60,63^
		- Inequitable access to advanced AI tools^59,62,64^
		- Bias and discrimination in data analysis outcomes^55,61^
7.	Damage to research quality and evaluation processes	- Increase in pseudoscience and invalid research^57,66^
		- Decline in trust toward published research^61,67,70^
		- Damage to the peer review system^60,67^
		- Editors’ and reviewers’ limited ability to detect machine-generated content^60,67^
8.	Risk of data security and confidentiality breaches	- Leakage of research data to LLMs^54,55,56,58,61^
		- Violation of research participants’ privacy^56^
		- Lack of researcher control over input data^55,56^
9.	Black-Box nature of LLMs	- Uncertainty regarding the sources of models’ training data^55,72^
		- Lack of transparency in how machine outputs are generated^55,57,72^
		- Researchers’ inability to assess the accuracy of machines’ decision-making processes due to the opacity of their internal mechanisms^54^
10.	Absence of binding policies	- Reliance on advisory or non-binding guidelines^55,70,71,72^
		- Lack of alignment between organizational and journal-level policies^55,56,62,71^
		- Absence of formal national mechanisms for oversight and accountability^61,71,72^
		- Multiplicity and inconsistency among international standards^71,72^

Abbreviations: LLM, large language model; AI, artificial intelligence. ^a^ Combination of AI and plagiarism.

 The most frequently reported challenges were: “Generation of fabricated data, evidence, and hallucinations without support from reliable sources” (frequency = 9), “Generation of biased outputs in text rewriting and publication” (frequency = 9), “Risk of machine-mediated plagiarism, commonly referred to as AIgiarism” (frequency = 7), and “Uncertainty regarding ownership of AI-generated scholarly content” (frequency = 7). Frequency indicates the number of studies reporting each theme.

###  Factors Influencing the Ethical Use of AI Tools in Research Publishing

 This scoping review identified ten main dimensions of factors influencing the ethical use of AI in research publishing. These factors include individual, institutional, and structural elements that affect both researcher behavior and the research system.

 The most frequently reported factors were: “Mandatory explicit and comprehensive disclosure of how AI tools are used across different stages of the research process” (frequency = 5) and “Development of integrated international guidelines to delineate ethical boundaries between proper use and misuse of AI tools” (frequency = 5) (Table 3). These findings suggest that the ethical application of AI in scholarly publishing necessitates a coordinated, multi-level approach involving individuals, institutions, and policy systems.

**Table 3:** Factors Affecting the Ethical Use of Artificial Intelligence Tools in Research Publication

**No.**	**Dimensions**	**Influencing Factors**
1.	Education aimed at enhancing ethical and digital literacy	- Education of researchers in ethical principles to promote the responsible use of AI tools^23,57,66^
		- Enhancement of researchers’ digital literacy to improve understanding of the limitations and errors of LLMs^54,65^
		- Training in effective prompt design to reduce errors and improve output accuracy^60,69^
		- Empowerment of researchers to critically evaluate AI-generated outputs^54,65^
		- Specialized training for journal editors and reviewers in detecting machine-generated texts^61,67^
2.	Human oversight and researcher accountability	- Mandatory human review of all AI-generated outputs to ensure ethical responsibility^55,60,63,65^
		- Emphasis on the researcher’s full accountability for the final manuscript^56,67,68,70^
		- Existence of formal mechanisms for correcting errors arising from the authorized use of AI tools^61,66^
3.	Promotion of ethical conduct in research culture	- Valuing transparency, accuracy, and adherence to publication ethics within research systems^23,72^
		- Strengthening a culture of scientific integrity in research involving AI tools^61,66,72^
		- Reducing publication-related pressures within educational and research systems^66,70^
		- Institutional modeling and support for responsible research grounded in ethical standards^61,71,72^
4.	Necessity of disclosure and traceability of AI contributions	- Mandatory explicit and comprehensive disclosure of how AI tools are used across different stages of the research process^55,62,66,68,70^
		- Documentation of interactions with AI models (including prompts and outputs) to support transparency and traceability^66,68,69^
		- Clear delineation between human and machine contributions in content generation^55,66^
		- Transparency regarding the sources and nature of the models’ training data^54,55^
5.	Data management and information security	- Prohibiting the input of sensitive data into public AI tools^54,55,56^
		- Anonymizing data before any authorized processing^57,61^
		- Transparency regarding how data is stored and reused by AI service providers^56,58^
6.	Development of quality control tools and mechanisms	- Development of monitoring tools to detect AI-generated text^54,55,66,69^
		- Creation of quality monitoring dashboards and model error assessment systems^60,66^
		- Regular monitoring of LLMs to ensure accuracy and validation^65^
7.	Equity in access to technology and resources	- Ensuring equal access to AI tools^59,62,64,70^
		- Supporting researchers from low-income countries through training and provision of free infrastructure^65,70^
		- Providing free or open-source tools to enhance research equity^62,70^
		- Improving language support for non-English-speaking researchers^57,65^
8.	Interdisciplinary collaboration and active stakeholder engagement	- Enhancing interactions among ethicists, engineers, publishers, and policy-makers to develop AI ethical standards^61,71,72^
		- Collaboration among universities, research centers, and industry to monitor ethical behavior in AI applications^62,72^
		- Sharing experiences and lessons learned across different disciplines^55,70^
		- Strengthening the link between technical standards and ethical principles^55,70^
9.	Formulation of coherent and integrated policies	- Development of integrated international guidelines to delineate ethical boundaries between proper use and misuse of AI tools^55,58,70,71,72^
		- Alignment of policies across universities, research institutions, and journals at the national level^55,56,62^
		- Emphasis on the enforceability of codes and guidelines^55,70,71,72^
		- Clear and consistent journal policies regarding disclosure of AI tool usage^55,62,64,70^
		- Highlighting the active role of editors and reviewers in monitoring responsible AI use^61,67^
10.	Continuous updating and monitoring of policies	- Continuous revision of guidelines in line with AI model developments^54,70,71^
		- Establishment of measurable indicators to assess the ethical use of AI^71^
		- Strengthening feedback loops for effective policy adjustments over time^70,71^

Abbreviations: LLM, large language model; AI, artificial intelligence.

## Discussion

 AI is a transformative force in research, but its increasing use has prompted significant ethical concerns, especially in publishing. The responsible application of AI in this domain necessitates balancing its technical benefits against social and ethical implications. This balance requires clear policies and enhanced user awareness. This study aimed to examine the key challenges and influencing factors related to the ethical use of AI in research publishing.

 Our findings highlight that integrating generative AI into scholarly workflows poses a significant threat to research originality. The studies demonstrate that LLMs can produce texts that evade traditional plagiarism detection systems. For instance, a study comparing ChatGPT-generated scientific abstracts with authentic ones found that the AI-generated summaries closely resembled original articles. Their linguistic quality and structural coherence made it difficult to determine the true source.^73^ Furthermore, our identification of compromised originality as a primary ethical concern aligns with the broader literature. For instance, research by Májovský et al confirms this finding. Language models are capable of generating fabricated and fraudulent articles that closely resemble human-written texts in terms of writing quality.^74^

 The evidence shows that generative AI fundamentally challenges ethical boundaries. It increases the likelihood of fabricated or inaccurate information entering academic publishing. Other systematic reviews suggest AI tools enhance efficiency and accuracy, but also introduce new misconduct threatening research integrity. Similarly, Chen and colleagues’ study highlights ethical concerns. It stresses the need for stronger academic ethical standards, improved researcher competence, and more rigorous review mechanisms, aligning with this study’s conclusions.^75^

 Our study identifies critical strategies for mitigating AI-related risks in scholarly publishing, including developing clear guidelines, providing targeted training for reviewers and editors, and disclosing AI tool usage. This finding aligns with the Knoechel et al study.^76^ Furthermore, Kumari et al introduced DEMASQ, a detector for ChatGPT-generated content. They demonstrated that this crisis requires active management,^77^ a path the present study also underscores as essential for the scholarly publishing system. Fabricated or manipulated texts and data pose a multifaceted technical challenge. This challenge undermines data and textual integrity, peer review trust, confidentiality, and process transparency.

 Data leakage and breaches of research confidentiality present significant ethical and security challenges with generative AI tools. As LLMs and generative AI are increasingly used for writing, editing, and analyzing scientific texts, concerns about research data security, confidentiality, and ownership have grown. Data entered into these chatbots may include raw research data, sensitive patient information, unpublished results, and peer-reviewed manuscripts. Disclosure of this data can lead to serious ethical and legal consequences.^78^

 Studies indicate data leakage in AI tools often stems from the technical nature of LLMs, not just conventional security vulnerabilities. These models may store user inputs for performance improvement and reuse them in training or processing. This raises the possibility that input data could be reproduced in responses to other users. Findings in scholarly publishing suggest some researchers, unaware of the consequences, input raw data or early drafts into public AI tools.^79^ To address these risks, frameworks like DataShield have been proposed. These ensure confidentiality and scientific transparency, aligning with this study’s emphasis on technical and legal mechanisms for data protection.^80^ Specifically, Giray and colleagues’ study examines AI use strengths, weaknesses, opportunities, and threats. It highlights that ChatGPT cannot replace human expertise, critical thinking, or peer review. The study emphasizes the necessity of human review for all AI-generated outputs to maintain ethical accountability, underscoring the importance of human oversight and researcher responsibility highlighted in this study.^60^

 Moreover, fair access to technology and the availability of free or open-source tools^70^ have been identified as essential for the ethical use of AI. Other core principles include transparency, privacy, accountability, and equity.^81^ Together, these principles highlight the researcher’s responsibility to review AI outputs, uphold ethical standards, and support equal access.

 More broadly, fostering a culture of research ethics is not optional. It is essential for maintaining public trust in science, ensuring the credibility of research findings, and sustaining knowledge production. The results of this study align with key international documents, including the Singapore Statement on Research Integrity and the European Code of Conduct for Research Integrity. These documents also emphasize that an ethical research culture is fundamental to public trust in science and the integrity of the knowledge production system.^82,83^

 As AI use in scientific publishing grows, clear reporting on how these tools are used is increasingly important for research integrity. The findings of this review show a lack of standardized reporting, which creates ambiguity in accountability. Emerging frameworks such as GAIDeT help address this gap by offering a practical way to support transparency.^44^ By standardizing what should be reported, these tools help journals distinguish between minor AI assistance and ethically sensitive contributions that require careful human validation. Integrating such tools into editorial workflows could improve the auditability of AI use and reduce misunderstandings about AI’s role in the research process. Further research should examine whether these taxonomies improve compliance and support research integrity across disciplines.

 Future studies should also explore the awareness, attitudes, and practices of researchers, reviewers, and editors regarding the ethical use of AI, using survey-based or qualitative methods. In addition, comparative studies are needed to assess the effectiveness of journal, university, and institutional policies and ethical guidelines on AI use in research and publishing.

###  Limitations

 This study has several limitations. First, limited access to some full-text articles may have affected the comprehensiveness of the review. Second, due to the novelty of the topic, many of the identified records were limited to letters to the editor or brief notes, with relatively few original research studies available. Third, there is potential for subjectivity inherent in the thematic analysis process. Furthermore, although the review protocol was developed a priori in accordance with methodological guidance, it was not publicly registered before the study. Finally, while this review identified and categorized the main ethical challenges and influencing factors, it did not aim to develop a fully integrated conceptual model. Further conceptual development and model validation will be undertaken in a subsequent complementary study using expert interviews.

## Conclusion

 AI in scientific publishing offers both important opportunities and significant challenges. Upholding principles such as research integrity, transparency, accountability, and respect for participants requires continuous training, clear policies, and effective oversight. The responsible use of AI requires a shift from ad hoc measures to an integrated ethical, technical, and legal governance system to prevent misuse and errors.

 This need is especially important in the health sciences, where published research informs clinical guidelines and health policy decisions. Clear standards for AI use in peer review and journal management are essential to protect the integrity of health evidence and maintain public trust. Strengthening ethical oversight of AI in publishing, therefore, supports reliable evidence for sound decision-making in health systems. By combining education, policy, and effective oversight, AI can be used safely and responsibly to improve the quality and credibility of scientific research.

## Acknowledgements

 The authors would like to sincerely thank all individuals who provided valuable comments and insights that contributed to the preparation of this manuscript.

## Disclosure of artificial intelligence (AI) use

 Not applicable.

## Ethical issues

 This study was approved by the Ethics Committee of Tehran University of Medical Sciences with code IR.TUMS.SPH.REC.1403.184.

## Disclaimer

 The views expressed in this article are those of the authors and do not necessarily reflect the official policy or position of any affiliated institution.

## Conflicts of interest

 Authors declare that they have no conflicts of interest.

## Supplementary files


Supplementary file 1. Detailed Database Search Strategies and Retrieval Results.
