# Supplementary material for: Ethical Use of Artificial Intelligence in Research Publication: A Scoping Review
Source: Int J Health Policy Manag. 2026 Aug 26;15:9759. doi: 10.34172/ijhpm.9759 (PMC13770245; doi:10.34172/ijhpm.9759)
Supplement: Supplementary file 1 [file ijhpm-15-9759-s001.pdf]

Article title: Ethical Use of Artificial Intelligence in Research Publication: A Scoping Review

Journal name: International Journal of Health Policy and Management (IJHPM)

Authors' information: Sara Dakhesh[1], Shahnaz Khademizadeh[2,1]*, Rasha Atlasi[3], Mojgan ZareiVenovel[4], Ali Ouchi[5]

[1]Department of Medical Library and Information Sciences, School of Allied Medical Sciences, Tehran University of Medical Sciences, Tehran, Iran.

[2]Department of Knowledge and Information Science, Faculty of Educational Sciences and Psychology, Shahid Chamran University of Ahvaz, Ahvaz, Iran.

[3]Endocrinology and Metabolism Research Center, Endocrinology and Metabolism Clinical Sciences Institute, Tehran University of Medical Sciences, Tehran, Iran.

[4]Social Determinants of Health Research Center, Lorestan University of Medical Sciences, Khorramabad, Iran.

[5]Department of Medical Library and Information Science, School of Management and Medical Informatics, Tabriz University of Medical Sciences, Tabriz, Iran.

***Correspondence to:** Shahnaz Khademizadeh; Email: s.khademi@scu.ac.ir

Citation: Dakhesh S, Khademizadeh S, Atlasi R, ZareiVenovel M, Ouchi A. Ethical use of artificial intelligence in research publication: a scoping review. Int J Health Policy Manag. 2026;15:9759. doi:10.34172/ijhpm.9759

Supplementary file 1. Detailed Database Search Strategies and Retrieval Results

No.	Database	Search strategy	Number of records retrieved
1.	Web of Science	**TS=((** (Intelligence AND Artificial) OR (Computer AND Reasoning) OR (Machine AND Intelligence)OR (Computational AND Intelligence) OR (Computer AND Vision AND System*) OR "Chat Bot" OR "Chat Bots" OR ChatBot OR ChatBots OR "AI Chat" OR GPT OR "Generative Artificial Intelligence" OR "Chat GPT" OR ChatGPT OR ChatGPTs **) AND (** (Fraud* AND Scientific) OR (Fraudulent AND Data) OR (Research AND Misconduct) OR (Scientific AND Dishonest*) OR (academic AND misconduct) **OR (Academic AND Integrity) OR (Research AND Integrity) OR (Scientific AND integrity) OR (Academic AND honesty**) **OR ((**Ethic* OR Moral OR misconduct OR **Integrity**) **AND** (Publish OR Publishing OR Publication* OR Writing* OR write OR draft* OR Composition OR	5897

		author* **OR Academic OR Student* OR Researcher* OR Professor* OR (Faculty AND member*) OR Publisher* OR Editor* OR Reviewer* OR Policymaker***) **)))**	
2.	Scopus	TITLE-ABS-KEY**((** (Intelligence AND Artificial) OR (Computer AND Reasoning) OR (Machine AND Intelligence) OR (Computational AND Intelligence) OR (Computer AND Vision AND System*) OR “Chat Bot” OR “Chat Bots” OR ChatBot OR ChatBots OR “AI Chat” OR GPT OR “Generative Artificial Intelligence“ OR “Chat GPT” OR ChatGPT OR ChatGPTs **) AND (** (Fraud* AND Scientific) OR (Fraudulent AND Data) OR (Research AND Misconduct) OR (Scientific AND Dishonest*) OR (academic AND misconduct) **OR (Academic AND Integrity) OR (Research AND Integrity) OR (Scientific AND integrity) OR (Academic AND honesty**) **OR ((**Ethic* OR Moral OR misconduct OR **Integrity**) **AND** (Publish OR Publishing OR Publication* OR Writing* OR write OR draft* OR Composition OR author* **OR Academic OR Student* OR Researcher* OR Professor* OR (Faculty AND member*) OR Publisher* OR Editor* OR Reviewer* OR Policymaker***))) **)**	10602
3.	PubMed	**(**"Artificial Intelligence"[Mesh] OR (Intelligence[TIAB] AND Artificial[TIAB]) OR (Computer[TIAB] AND Reasoning[TIAB]) OR (Machine[TIAB] AND Intelligence[TIAB]) OR (Computational [TIAB] AND Intelligence[TIAB]) OR (Computer [TIAB] AND Vision[TIAB] AND System*[TIAB]) OR “Chat Bot” [TIAB] OR “Chat Bots” [TIAB] OR ChatBot[TIAB] OR ChatBots[TIAB] OR “AI Chat” [TIAB] OR GPT [TIAB] OR “Generative Artificial Intelligence“[TIAB] OR "Generative Artificial Intelligence"[Mesh] OR “Chat GPT” [TIAB] OR ChatGPT[TIAB] OR ChatGPTs[TIAB]) **AND (**"Scientific Misconduct"[Mesh] OR (Fraud*[TIAB] AND Scientific[TIAB]) OR (Fraudulent [TIAB] AND Data[TIAB]) OR (Research[TIAB] AND Misconduct[TIAB]) OR (Scientific[TIAB] AND Dishonest*[TIAB]) OR (academic [TIAB] AND misconduct[TIAB]) **OR (Academic**[TIAB] **AND Integrity**[TIAB] **) OR (Research**[TIAB] **AND Integrity** [TIAB]) **OR (Scientific**[TIAB] **AND integrity**[TIAB] **) OR (Academic** [TIAB] **AND honesty**[TIAB] **) OR ((**"Ethics"[Mesh] OR Ethic*[TIAB] OR Moral[TIAB] OR misconduct[TIAB] OR **Integrity**[TIAB]) **AND** (Publish [TIAB] OR Publishing[TIAB] OR Publication*[TIAB] OR Writing*[TIAB] OR write[TIAB] OR draft*[TIAB] OR Composition[TIAB] OR Code*[TIAB] OR Reasearch*[TIAB] OR author*[TIAB] **OR Academic**	2245

		[TIAB] **OR Student*** [TIAB] **OR Researcher***[TIAB] **OR Professor***[TIAB] **OR (Faculty** [TIAB] **AND member***[TIAB] **) OR Publisher***[TIAB] **OR Editor***[TIAB] **OR Reviewer***[TIAB] **OR Policymaker***[TIAB])) **)**	
4.	Embase	("artificial intelligence"/exp OR (Intelligence:ti,ab,kw AND Artificial:ti,ab,kw) OR (Computer:ti,ab,kw AND Reasoning:ti,ab,kw) OR (Machine:ti,ab,kw AND Intelligence:ti,ab,kw) OR (Computational:ti,ab,kw AND Intelligence:ti,ab,kw) OR (Computer:ti,ab,kw AND Vision:ti,ab,kw AND System*:ti,ab,kw) OR "Chat Bot":ti,ab,kw OR "Chat Bots":ti,ab,kw OR ChatBot:ti,ab,kw OR ChatBots:ti,ab,kw OR "AI Chat":ti,ab,kw OR GPT:ti,ab,kw OR "Generative Artificial Intelligence":ti,ab,kw OR "generative artificial intelligence"/exp OR "Chat GPT":ti,ab,kw OR ChatGPT:ti,ab,kw OR ChatGPTs:ti,ab,kw) **AND** ("scientific misconduct"/exp OR (Fraud*:ti,ab,kw AND Scientific:ti,ab,kw) OR (Fraudulent:ti,ab,kw AND Data:ti,ab,kw) OR (Research:ti,ab,kw AND Misconduct:ti,ab,kw) OR (Scientific:ti,ab,kw AND Dishonest*:ti,ab,kw) OR (academic:ti,ab,kw AND misconduct:ti,ab,kw) **OR (Academic**:ti,ab,kw **AND Integrity**:ti,ab,kw **) OR (Research**:ti,ab,kw **AND Integrity**:ti,ab,kw) **OR (Scientific**:ti,ab,kw **AND integrity**:ti,ab,kw**) OR (Academic**:ti,ab,kw **AND honesty**:ti,ab,kw) **OR** (("ethics"/exp OR Ethic*:ti,ab,kw OR Moral:ti,ab,kw OR misconduct:ti,ab,kw OR **integrity**:ti,ab,kw) **AND** (Publish:ti,ab,kw OR Publishing:ti,ab,kw OR Publication*:ti,ab,kw OR Writing*:ti,ab,kw OR write:ti,ab,kw OR draft*:ti,ab,kw OR Composition:ti,ab,kw OR author*:ti,ab,kw **OR Academic**:ti,ab,kw **OR Student***:ti,ab,kw **OR Researcher***:ti,ab,kw **OR Professor***:ti,ab,kw **OR (Faculty**:ti,ab,kw **AND member***:ti,ab,kw **) OR Publisher***:ti,ab,kw **OR Editor***:ti,ab,kw **OR Reviewer***:ti,ab,kw **OR Policymaker***:ti,ab,kw)))	2218
